# Purulent pericarditis due to co-infection with *Streptococcus pneumoniae *and *Mycobacterium tuberculosis *in a patient with features of advanced HIV infection

**DOI:** 10.1186/1471-2334-7-12

**Published:** 2007-03-08

**Authors:** Andrew Louw, Mohammed Tikly

**Affiliations:** 1 Department of Medicine, Chris Hani Baragwanath Hospital, Soweto, South Africa; 2 University of the Witwatersrand, Johannesburg, South Africa

## Abstract

**Background:**

Both *Mycobacterium tuberculosis *and *Streptococcus pneumoniae *are common pathogens in patients with HIV infection.

**Case Presentation:**

We present an unusual case of purulent pericarditis resulting in cardiac tamponade due to infection with both organisms. We highlight the re-emergence of pneumococcal pericarditis in the HIV era and describe the pitfalls and challenges in the diagnosis of this condition.

**Conclusion:**

Clinicians working in HIV endemic areas need to consider dual infection with these organisms in patients who respond inadequately to either antibiotics or anti-tuberculous therapy alone.

## Background

*Streptococcus pneumoniae *is a common cause of infections of the middle ear and airways in man. In the pre-antibiotic era, haematogenous spread to more distant sites, termed invasive pneumococcal infection (IPI), was a major cause of morbidity and mortality. In recent years, a resurgence of IPI has been observed in patients with underlying human immunodeficiency (HIV) infection [1,2]. We describe a case of IPI, presenting as cardiac tamponade due to purulent pericarditis, in a patient co-infected with tuberculosis (TB) and clinical features of advanced HIV infection.

## Case history

A 33 year old man presented with a 3 week history of a cough productive of white sputum, pleuritic type chest pain and night sweats. More recently he had developed swollen feet and New York Heart Association class III dyspnoea. He had been experiencing chronic weight loss and generalised body weakness. He had no known TB contacts and had no significant past medical history.

On admission, the patient was apyrexial, pale, dyspnoeic at rest, with a blood pressure of 113/88 mmHg, a palpable pulsus paradoxus and pulse rate 122 beats per minute. There were signs of biventricular failure including a raised jugular venous pressure, bibasal crackles, right pleural effusion, 8 cm firm hepatomegaly and bilateral pedal oedema up to the knees. Examination of the precordium revealed a poorly palpable apex beat with soft heart sounds and a gallop rhythm. General examination revealed obvious stigmata of HIV infection, including wasting, oral candidiasis, melanonychiae (blue nails) and generalised lymphadenopathy, including epitrochlear nodes.

**Figure 1:**
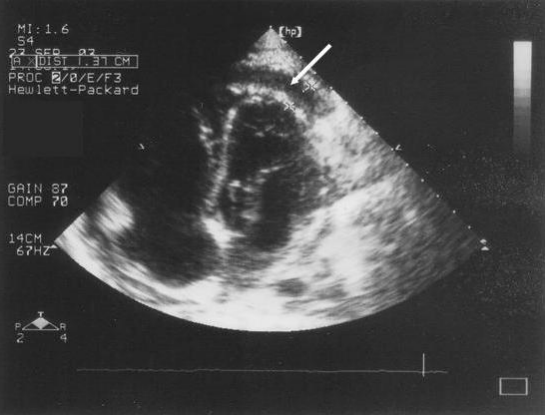
Echocardiogram showing apical collection of fluid in the pericardial space (white arrow).

Chest x-ray on admission showed a massively enlarged globular cardiac shadow and large right pleural effusion. The electrocardiogram revealed a sinus tachycardia, small QRS complexes and inverted T-waves in the precordial leads. Initial laboratory investigations showed a normal total white cell count of 7.17 × 10^9^/l, with an absolute neutrophilia of 5.43 × 10^9^/l, haemoglobin of 9.1 g/dl and platelet count of 327 × 10^9^/l. His serum urea was mildly elevated at 8.2 mmol/l with normal electrolytes. The patient refused to consent to testing for HIV infection despite repeated counselling. An echocardiogram performed on the day after admission demonstrated a massive pericardial effusion with fibrin strands and right ventricular systolic collapse, features consistent with a diagnosis of cardiac tamponade (Fig. 1). Pericardiocentesis was performed and 950 ml of brown, purulent fluid drained.

Despite empiric 4 drug anti-tuberculous therapy (rifampicin, isoniazid, pyrazinamide and ethambutol), oral prednisone (40 mg bd) and furosemide (40 mg bd), the patient's condition worsened and he became pyrexial. Four days post-admission, a penicillin-resistant strain of *Streptococcus pneumoniae *(MIC = 0.5 ug/ml) was isolated from the pericardial aspirate, but no acid fast bacilli were observed on auramine stain. The patient's clinical condition improved marginally on intravenous cefotaxime, but 10 days post-admission a repeat echocardiogram showed re-accumulation of the pericardial fluid with collapse of the right ventricle. At this point a subxiphoid pericardiotomy was performed and a pericardial drain inserted. The patient developed a left sided pleural effusion post-operatively and a chest drain was inserted. A follow-up echocardiogram showed a thickened pericardium a small pericardial collection (<0.5 cm), but with no evidence of tamponade.

Because of an ongoing pyrexia and chest symptoms, chest ultrasonagraphy was ordered which revealed bilateral loculated effusions. A chest CT scan showed supraclavicular, mediastinal and abdominal adenopathy; residual pericardial thickening and effusion; bilateral residual pleural effusions; and patchy consolidation of the right upper lobe. At this stage the patient refused further surgical intervention and was discharged on anti-tuberculous therapy. Culture of pericardial fluid subsequently grew *Mycobacterium tuberculosis*.

## Discussion

Historically, purulent pericarditis was seen most commonly as a complication of pneumonia in otherwise healthy children and young adults [3,4]. With the advent of antibiotics for widespread use in 1943, the condition became rare and was confined mainly to the elderly with serious co-morbidity. In more recent times, two cases of HIV-1 positive, female patients with TB who developed pneumococcal pericarditis have been reported in the United States [5]. Our case represents an additional example of purulent pneumococcal pericarditis in a patient with underlying HIV infection, although the latter could not be definitively proven. To the best of our knowledge, there are no published reports of co-infection with TB and pneumococcus of the pericardium from Africa. However, Schleicher and Feldman have reported nine cases of dual infection with these organisms presenting as community acquired pneumonia in patients with HIV infection [6].

In most cases, purulent pneumococcal pericarditis is a result of either direct spread or bacteraemia due to intrathoracic diseases such as pneumonia, empyema, myocardial abscesses or endocarditis [3,4]. The pericardium is rarely a source of primary infection. A pre-existing aseptic pericarditis is a predisposing condition in nearly half of cases [3]. The clinical diagnosis of pneumococcal pericarditis is often challenging and critical to make, since the condition is fatal if left untreated [4]. In large studies, ante-mortem diagnosis has been made, at most, in only a fifth of all cases [3,4]. Pulsus paradoxus, where present, has been found to be a useful diagnostic sign [4], which was evident in our patient.

Specific defects in host defence, particularly humoral immunity make HIV-1 seropositive individuals particularly susceptible to infection with encapsulated bacteria [2]. Contrary to the common notion, *Streptococcus pneumoniae *and not *Mycobacterium tuberculosis *is the commonest and earliest serious pathogen to cause disease during HIV infection [1]. Furthermore, HIV infection predisposes to penicillin-resistant strains, as in our case, which are otherwise seen only children [7].

Tuberculosis is a common cause of pericarditis in HIV-endemic areas and there is indirect evidence to suggest that the outcome of tuberculous pericarditis is worse in patients with underlying HIV infection. A recent large multicentre study of tuberculous pericarditis from sub-Saharan Africa has shown that, compared to otherwise immunocompetent patients, patients with clinical HIV infection have a greater degree of dyspnoea, haemodynamic instability, cardiomegaly and electrocardiographic features of myopericarditis [8].

In conclusion, this case illustrates that while TB remains the commonest infectious cause of pericardial effusion in sub-Saharan Africa, in the context HIV infection, pneumococcal pericarditis needs to be actively excluded. Particular attention to the cardiovascular examination should be given in HIV positive patients with pneumococcal pneumonia or empyema.

## Competing interests

The author(s) declare that they have no competing interests.

## Authors' contributions

AL and MT both contributed to the clinical work-up of the patient and writing-up of the manuscript.

## Pre-publication history

The pre-publication history for this paper can be accessed here:


